# Supplementary material for: COVID-19 Racism and Chinese American Families’ Mental Health: A Comparison between 2020 and 2021
Source: Int J Environ Res Public Health. 2023 Apr 7;20(8):5437. doi: 10.3390/ijerph20085437 (PMC10138552; doi:10.3390/ijerph20085437)
Supplement: Supplementary file 1 [file ijerph-20-05437-s001.zip › ijerph-2274658-supplementary.pdf]

Supplement 1

Among the 2020 sample, 230 parents (43% of the 2020 parent sample) and 57 dyads (25% of the 2020 dyad sample) participated in 2020, and 299 parents (57% of the 2020 parent sample) and 168 dyads (75% of the 2020 dyad sample) participated in both 2020 and 2021. The 2021 sample included: (1) the sample who participated in both 2020 and 2021 and 216 (42% of the 2021 parent sample) parents and 148 dyads (47% of the 2021 dyad sample) who participated only in 2021. We compared the demographic characteristics of the parent and dyad samples who participated in 2020 only, in 2021 only, and in both 2020 and 2021. For the parent sample, those who participated in both years reported being older, having higher levels of education, and coming from households of higher socio-economic status (SES) compared to parents who participated in 2020 only and parents who participated in 2021 only; no differences were found for other demographic variables across the three groups. In the dyad samples, parents who participated in 2021 only reported being younger and coming from households of lower SES than the other two groups; no other differences were found across the three groups. For the dyad sample who participated in both 2020 and 2021, demographic information from 2020 was used when conducting the comparisons.

Supplement 2

Cutoffs for BDI-II: slightly to moderately elevated risk scores 14 - 27; substantial risk scores 28 or above.[1] Cutoffs for GAD-7: slightly elevated risk scores 10 - 14; substantial risk scores 15 or above.[2] Cutoffs for SDQ: slightly elevated risk scores 12 - 15; substantial risk scores 16 or above.[3]

Supplement 3

Cheah, C.S.L.; Wang, C.; Ren, H.; etc. COVID-19 racism and mental health in Chinese American Families. *Pediatrics* 2020, *146*, e2020021816. doi:10.1542/peds.2020-021816

How often has these things happened during the COVID-19 pandemic? Please rate your answer on a 5-point scale:

**Online Direct Discrimination**	**1**<br>**Never**	**2**<br>**Once or twice a month**	**3**<br>**A few times a month**	**4**<br>**Once or twice a week**	**5**<br>**Almost everyday**
1. People have said mean or rude things about me because of my race or ethnic group online.					
2. People have excluded me from a site because of my race or ethnic group online.					
3. People have threatened me online with violence because of my race or ethnic group.					
4. People have shown me a racist image online.					
**Online Vicarious Discrimination**					
1. People have cracked jokes about people of my race or ethnic group online.					
2. People have said things that were untrue about people in my race or ethnic group online.					
3. I have witnessed people saying mean or rude things about another Asian person online.					
**In-person Direct Discrimination**					
1. Some people were unfriendly or unwelcoming toward me because of my Chinese background.					
2. Some people didn't want to be with me because of my Chinese background.					
3. Some people avoided me because of my Chinese background.<br>4. Some people made fun of me or Chinese people.					
5. Some people were rude to me because of my Chinese background.					

**In-person Vicarious Discrimination**	**1**<br>**Never**	**2**<br>**Once or twice a month**	**3**<br>**A few times a month**	**4**<br>**Once or twice a week**	**5**<br>**Almost everyday**
1. Someone said something negative about Chinese people (for example, their diet) related to the COVID-19 outbreak.					
2. Someone said something about avoiding places with Chinese people because of the COVID-19 outbreak.					
3. Someone tried to find out if you or your family has been to China since the COVID-19 outbreak.					
4. People have said things that were untrue about Chinese people because of the COVID-19 outbreak.					

Please indicate your agreement with the following items using this rating scale:

**Health-related Sinophobia**	**1**<br>**Strongly Disagree**	**2**<br>**Disagree**	**3**<br>**Neutral**	**4**<br>**Agree**	**5**<br>**Strongly Agree**
1. A lot of Americans are afraid that Chinese people are going to make American sick.					
2. A lot of Americans consider Chinese people as a threat to public health in America.					
**Media Sinophobia**					
1. The U.S. media presents Chinese people as dangerous.					
2. Chinese people and culture are presented as a threat to American culture in the media.					
3. U.S. media spreads a lot of fear about China and Chinese people.					
4. U.S. media represents Chinese people as a threat to public health.					
5. Chinese people and culture are presented as a threat to American public health in the media.					

Thinking about your thoughts, feelings, and behaviors around the COVID-19 (coronavirus) illness, please answer the following questions:

**Government-related Worries/Fears**	**1 Strongly Disagree**	**2 Somewhat Disagree**	**3 Somewhat Agree**	**4 Strongly Agree**
I am concerned that the government's response to the COVID-19 outbreak (e.g., blaming China, calling it the "China virus" or "Wuhan Virus") will make life more difficult for Chinese Americans				
I am worried that Chinese Americans will be targeted because of the government's handling of the COVID-19 outbreak.				
I am afraid for my safety as a Chinese American because of how the government is labelling the COVID-19 virus as "China virus" or "Wuhan Virus"				

References

1. Beck AT, Steer RA, Brown GK. Beck depression inventory-II. *San Antonio*. 1996;78(2):490-498.

2. Spitzer RL, Kroenke K, Williams JBW, Lowe B. A brief measure for assessing generalized anxiety disorder: the GAD-7. *Arch Intern Med*. 2006;166(10):1092-1097. doi:10.1001/archinte.166.10.1092

3. Bourdon KH, Goodman R, Rae DS, Simpson G, Koretz DS. The strengths and difficulties questionnaire: US normative data and psychometric properties. *J Am Acad Child Adolesc Psychiatry*. 2005;44(6):557-564. doi:10.1097/01.chi.0000159157.57075.c8

4. Cheah, C.S.L.; Wang, C.; Ren, H.; etc. COVID-19 racism and mental health in Chinese American Families. *Pediatrics* 2020, *146*, e2020021816. doi:10.1542/peds.2020-021816
